# A rare presentation of Takayasu’s arteritis- unilateral finger clubbing – case report

**DOI:** 10.1186/s41927-020-00166-z

**Published:** 2020-12-10

**Authors:** Shania Niromi Gunasekera, Chirath Madurapperuma, Nilusha Weerasooriya, Harindra Karunathilake, Ananda Jayanaga

**Affiliations:** National Hospital of Sri Lanka, Colombo 08, Sri Lanka

**Keywords:** Takayasu’s arteritis, Large vessel vasculitis, Unilateral clubbing, Case report

## Abstract

**Background:**

Takayasu’s arteritis (TA) is a granulomatous, large vessel vasculitis with a preponderance for young women. The inflammation results in disruption of the arterial endothelium causing stenosis, endoluminal thrombosis and aneurismal dilatation. Early disease presentation is with nonspecific general symptoms, and in such instances, the diagnosis can be missed. Unilateral clubbing is a manifestation of myriad of diseases, but is not a common sign of TA. In medical literature, only three such cases have been reported.

**Case presentation:**

We present a 24-year-old female who presented with multiple constitutional symptoms such as arthralgia, malaise, poor appetite and two episodes of syncope over 3 months’ duration. On examination, unilateral finger clubbing was observed in the right hand, with very low volume radial, ulnar and brachial artery pulses on the ipsilateral side. Her blood pressure measured on the unaffected arm, was normal. Inflammatory markers were elevated and magnetic resonance angiogram (MRA) confirmed TA.

**Conclusion:**

Although rare, unilateral clubbing may be a manifestation of TA. Therefore, detection of unilateral clubbing should raise a strong clinical suspicion of TA and prompt early diagnosis and initiation of treatment.

## Background

Takayasu’s arteritis (TA) is a granulomatous arteritis involving large vessels, predominantly the aorta and its major branches [1]. Inflammation of the arteries causing endothelial damage can lead to vessel wall thickening, vascular occlusion and thrombus formation [2]. The resultant ischemia may cause disabling symptoms. Early in the disease, symptoms are nonspecific, such as fever, fatigue, arthralgia, myalgia and weight loss, and in such instances, the diagnosis is often missed [2].

We present a case of Takayasu’s arteritis, presenting with constitutional symptoms and unilateral finger clubbing. Only three similar cases have been reported in the literature [3–5].

## Case presentation

A 24-year-old female from Southern province of Sri Lanka, presented with arthralgia, malaise and poor appetite for 3 months’ duration. A week prior to the admission, she had an episode of transient loss of consciousness, which was followed by an uneventful recovery. It was assumed to be a vasovagal syncope, since the semiology was not suggestive of a seizure and it was not associated with chest pain or palpitations. She had a similar event a month ago, which also resolved spontaneously upon lying down.

She complained of arthralgia involving all small and large joints indiscriminately without any signs of inflammation. She did not give a history of fever, chills or rigors, drenching night sweats or weight loss. She denied cough, difficulty in breathing, chest pain, headache, visual disturbances, photophobia, or photosensitive rashes. She also did not have cold intolerance, constipation, menorrhagia, polyuria or polydipsia. She denied neck pain, limb claudication or Raynaud’s phenomenon. She was not on any medications. She had regular menstrual periods. She had no premorbid illnesses and her family history was unremarkable.

**Fig. 1:**
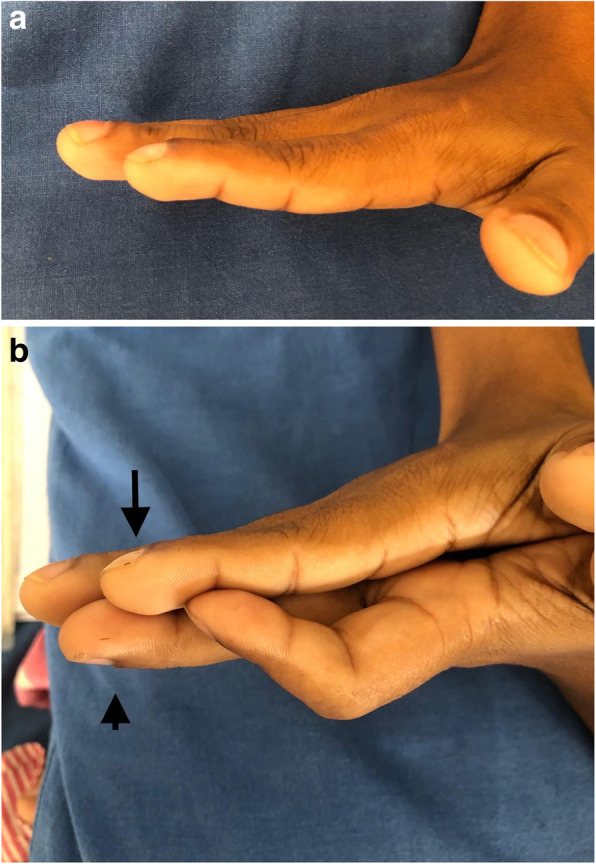
**a** Right hand finger clubbing. **b** – right side unilateral finger clubbing (arrow)with sparing of left hand fingers (arrow head)

On examination, her height was 144 cm and she weighed 42 kg with a body mass index of 21 kg/m2. She was not febrile or pale and there was no hair loss, rashes, oral or genital ulcers. She did not have redness in the eyes. She had grade 2 clubbing involving the fingers of the right hand (Fig. 1a, b). Interestingly, clubbing was absent in the fingers of the left hand and toes of both feet. Her radial, ulnar and brachial pulses were barely felt in the right arm, whereas in the left arm, all the pulses were felt with normal volume. Blood pressure was not recordable in the right arm and in the left, it was 112/88 mmHg. The carotid and femoral pulses were palpable in normal volume and there was no radio-radial or radio-femoral delay. Mild tenderness was elicited over the right carotid artery but, there was no bruit. Cardiac examination was normal. Lung fields were clear. Rest of the examination was unremarkable.

Full blood count revealed neutrophil leukocytosis, mild normocytic normochromic anemia and thrombocytosis. Her erythrocyte sedimentation rate (ESR) was > 110 mm/ 1st hour and C-reactive protein (CRP) was 24 mmol/l. Liver and renal functions, serum electrolytes including calcium and magnesium, and thyroid functions were normal. The chest and spine X-ray images failed to show any abnormalities. Computed tomography (CT) aortogram revealed thrombosis of brachio-cephalic trunk, right common carotid, subclavian and axillary arteries.

Her clotting studies, including prothrombin time, thrombin time and activated partial thrombin time were normal. Anti-thrombin iii, Protein C and Protein S levels were normal, thereby excluding common diseases of thrombophilia. Anti-cardiolipin IgM antibody, Lupus anticoagulant, anti Beta 2 glycoprotein IgG and IgM antibodies were negative. Anti nuclear antibody (ANA), anti double stranded DNA (anti ds DNA), cytoplasmic anti neutrophil cytoplasmic antibodies (ANCA) and perinuclear ANCA, were all negative.

**Fig. 2:**
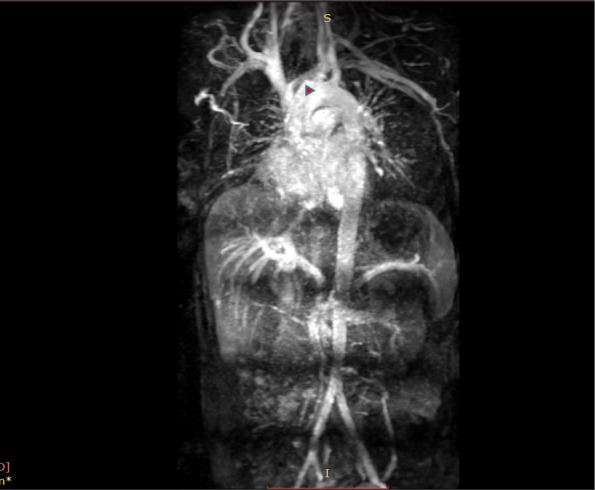
MR angiogram of aorta and its branches - the brachio-cephalic trunk is obliterated at it’s origin (arrow head)

Magnetic Resonance angiogram (MRA) was performed to further delineate the cause for thrombosis. In the MRA, brachio-cephalic artery was completely occluded at it’s origin. There were collateral vessels, formed distal to the brachio cephalic stump (Fig. 2).

Considering the clinical presentation, elevated inflammatory markers and MR angiographic evidence, TA involving the brachio-cephalic artery and its branches was diagnosed.

She was given intravenous methylprednisolone 500 mg daily for 3 days followed by oral prednisolone 40 mg daily. Oral Azathioprin 50 mg daily was added as a steroid sparing agent. She was anticoagulated, initially with subcutaneous enoxaparin followed by warfarin 4 mg, daily.

After starting the treatment, her symptoms subsided and 3 months in to the therapy, clubbing still persisted. ESR reduced to 35 and she is currently being followed up monthly, at our clinic.

## Discussion

TA is an autoimmune, chronic, granulomatous vasculitis involving the large arteries. Inflammation of the endothelium causes stenosis, thrombosis, luminal occlusion and aneurysmal dilatation [2]. TA has a preponderance for females, accounting for over 90% of the cases. The incidence of TA is highest in the fourth decade [6]. The etiology of TA has not been fully understood but a genetic basis has been described. Certain infections, particularly *Mycobacterial tuberculosis* has been linked to the pathogenesis of TA [1].

Early symptoms include headaches, fever, weight loss, myalgia, and arthralgia. Hypertension is common, which occurs in 80% of cases due to stenosis of the renal arteries resulting in activation of renin-angiotensin- aldosterone system [1]. Hypertension was absent in this patient, probably because of her early presentation before the renal arteries were involved. The classic features of TA such as limb claudication, carotid or subclavian arterial bruits and end organ ischemia, which usually occur as the disease progresses, were absent in this patient.

Markers of inflammation such as ESR and CRP are elevated in most, but not all patients with TA. The elevated CRP level correlates with thrombotic events [1].

European league against Rheumatism (EULAR) recommends MR angiogram as the first imaging modality to detect vessel wall inflammation and changes [7]. Positron emission tomography (PET) scan, when combined with CT shows diffuse hypermetabolism of the involved arteries and enables evaluation of wall thickness and luminal changes. However, its cost, availability and exposure to high dose of radiation limit its use [8].

The index case fulfilled 4 out of 6 ACR criteria (Table 1). In the initial stages nonspecific symptoms predominate. Hence, the diagnosis is often missed. In our patient, the initial clue to the diagnosis was unilateral finger clubbing. There are only three similar cases reported in the literature [3–5].

**Table 1:** The diagnostic criteria for TA coined by American College of Rheumatology (ACR) [9]

The diagnosis is most likely when three or more out of the following six criteria are met	
I	age of onset younger than 40 years
II	claudication of extremities
III	decreased pulsation of one or both brachial arteries
IV	difference of systolic pressure between arms at least by 10 mmHg
V	bruits over one or both subclavian arteries or the abdominal aorta
VI	arteriography showing narrowing or occlusion of the entire aorta, its primary branches, or large arteries in the proximal upper or lower extremities not cause by arteriosclerosis, fibromuscular dysplasia, or other causes

Finger clubbing can be associated with a multitude of diseases and unilateral finger clubbing has been reported with hemiplegia, dialysis fistulae, arteritis of the large arteries, and ulna artery aneurysm [10]. The pathogenesis of digital clubbing is best explained by the hypothesis put foreword by Dickinson and Martin [11]. They proposed that, when unfragmented megakaryocytes enter the systemic circulation through A-V malformations, their large size make them stagnate in the fingertip circulation causing local tissue hypoxia. This in turn induces the release of platelet-derived growth factor (PDGF), causing increased vascular permeability, number of vascular smooth muscle cells and fibroblasts [11]. Atkinson and Fox proposed that vascular endothelial growth factor (VEGF) and hypoxia inducible factor (HIF), which are released in response to tissue hypoxia, also played a synergistic role with PDGF, in the pathogenesis of digital clubbing [12]. Though this hypothesis does not entirely explain the unilateral finger clubbing in our patient, severe arteritis with rapidly progressive stenosis and the resultant local hypoxia would have triggered the aforementioned pathological process. Clubbing, at its early stage may reverse with treatment of the causative disease, but not so if it has become chronic [10].

The EULAR 2018 guideline for the management of large-vessel vasculitis recommends initiation of high dose corticosteroid therapy at the point of diagnosis. A steroid sparing immunosuppressant should be added if corticosteroids alone fail to achieve and maintain remission. Tocilizumab, a TNF –alfa inhibitor can be considered in treating recalcitrant disease. Regular antiplatelet or anticoagulation is indicated when organ threatening ischemia is evident. In our patient, anticoagulation was commenced as she had a large thrombus occluding the right common carotid artery, which may have been the cause for the two episodes of syncope.

Disease monitoring is done with periodic evaluation of clinical symptoms, markers of inflammation and imaging. The angiographic abnormalities do not disappear, but stop their progression with treatment [13].

TA is a chronic disease with a relapsing course [14]. Although the symptoms improve with glucocorticoid therapy, they tend to relapse once the dose is tapered off. Relapses have continued to occur in at least 50% of patients despite adjuvant immunosuppressive therapies or surgical revascularizations [6, 15].

TA is a granulomatous large vessel vasculitis with nonspecific symptoms at the outset, which can be easily overlooked. Unilateral clubbing is an extremely rare manifestation of TA. Therefore, detection of unilateral clubbing should raise a strong clinical suspicion of TA, with prompt diagnosis and initiation of treatment.

## Data Availability

Not applicable.

## Abbreviations

TA: Takayasu’s arteritis
ESR: Erythrocyte sedimentation rate
CRP: C- reactive protein
MRA: Magnetic resonance aortogram
ANA: Anti nuclear antibody
dsDNA: Double stranded DNA
pANCA: Perinuclear anti neutrophil cytoplasmic antibody
cANCA: Cytoplasmic anti neutrophil cytoplasmic antibody
PET: Positron emission tomography
PDGF: Platelet derived growth factor
VEGF: Vascular endothelial growth factor
HIF: Hypoxia inducible factor
